# B cell development: transcriptional regulation and immunological mechanisms in homeostasis

**DOI:** 10.3389/fimmu.2025.1593338

**Published:** 2025-08-08

**Authors:** Jorge Gómez-Manríquez, Jorge Hernández-Bello, José Francisco Muñoz-Valle, Sonia Sifuentes-Franco, Omar Graciano-Machuca, José Javier Morales-Núñez

**Affiliations:** ^1^ Programa Educativo Médico Cirujano y Partero, Centro Universitario de Ciencias de la Salud (CUCS), Universidad de Guadalajara, Guadalajara, Jalisco, Mexico; ^2^ Instituto de Investigación en Ciencias Biomédicas (IICB), Departamento de Biología Molecular y Genómica, Centro Universitario de Ciencias de la Salud, Universidad de Guadalajara, Guadalajara, Jalisco, Mexico; ^3^ Laboratorio de Ciencias Clínicas, Departamento de Ciencias de la Salud, Centro Universitario de los Valles, Universidad de Guadalajara, Ameca, Jalisco, Mexico; ^4^ Departamento de Morfología, Centro Universitario de Ciencias de la Salud, Universidad de Guadalajara, Guadalajara, Jalisco, Mexico

**Keywords:** B lymphocyte, B cell receptor, transcriptional factors, tolerance, immunoglobulins

## Abstract

B lymphocytes are essential elements of the adaptive immune response, performing critical functions such as antigen presentation, cytokine secretion, and antibody production. Their development follows a tightly regulated progression from hematopoietic stem cells to differentiated plasma or memory cells, orchestraeted by key transcriptional factors including *PU.1, Ikaros, E2A, Pax-5*, and *BCL6.* These factors govern gene expression essential for processes such as V(D)J recombination, somatic hypermutation, and immunoglobulin class switching—ensuring proper lineage commitment and the maintenance of immunological tolerance. Dysregulation of these pathways, whether through genetic or epigenetic alterations or chronic inflammatory stimuli, can result in autoimmunity, persistent inflammation, or B cell malignancies. This review provides a comprehensive analysis of the transcriptional and immunological mechanisms underlying B cell development and homeostasis, emphasizing their roles in disease pathophysiology and potential as therapeutic targets.

## 1 Introduction

**Figure 1:**
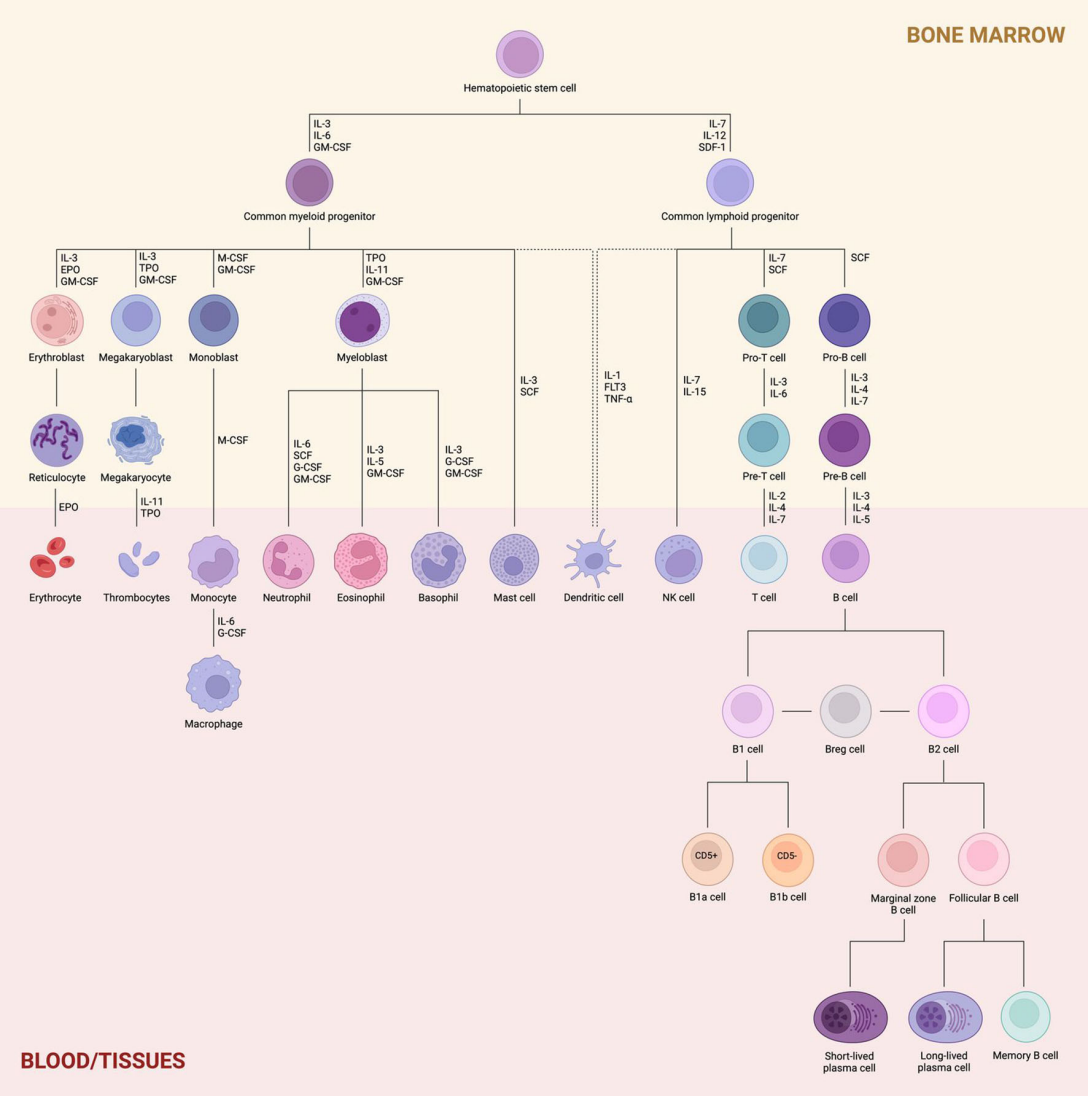
Hematopoiesis and lymphoid lineage. Proliferating hematopoietic stem cells (HSCs) differentiate into either common myeloid progenitors (CMPs) or common lymphoid progenitors (CLPs), with lineage-specific commitment regulated by key transcriptional factors. The myeloid lineage gives rise to erythroblasts, megakaryoblasts, monoblasts, and myeloblasts within the bone marrow, which then mature into erythrocytes, thrombocytes, monocytes/macrophages, and granulocytes (neutrophils, eosinophils, and basophils), respectively, once they migrate to the bloodstream or peripheral tissues. Mast cells and dendritic cells may also derive from this lineage. In contrast, the lymphoid lineage produces T and B cell precursors (pro- and pre-T/B cells) in the bone marrow. Upon migration to the periphery, these precursors differentiate into mature T and B cells, and also generate NK cells and dendritic cells. B cells are further subdivided into B1 and B2 cells. B1 cells differentiate into B1a and B1b subsets based on CD5 surface expression, while B2 cells give rise to marginal zone and follicular B cells, depending on their anatomical niche. Marginal zone B cells predominantly form short-lived plasma cells, whereas follicular B cells contribute to long-lived plasma cells and memory B cells.

Hematopoiesis is a dynamic process that generates approximately 500 billion new blood cells daily. It is traditionally divided into two main lineages: myeloid and lymphoid. The myeloid lineage supports innate immunity, oxygen transport, and hemostasis, while the lymphoid lineage is primarily responsible for adaptive immunity (1). The classical model of hematopoiesis describes a hierarchical organization that begins with hematopoietic stem cells (HSCs) in the bone marrow. These cells either undergo self-renewal or differentiate into multipotent progenitors (MPPs), which lack self-renewal capacity but retain the potential to generate all blood cell types through successive bifurcations (2). This differentiation pathway includes the divergence into common myeloid progenitors (CMPs) and common lymphoid progenitors (CLPs), ultimately giving rise to granulocytes, monocytes, erythrocytes, megakaryocytes, T cells, B cells, and natural killer (NK) cells. Both lineages are also capable of generating dendritic cells (DC), as illustrated in **Figure 1** (3).

However, recent advances challenge the oversimplification inherent in this hierarchical model and instead support a continuum model of hematopoiesis. In this revised framework, hematopoietic stem and progenitor cells (HSPCs) gradually acquire lineage-specific programs without transitioning through clearly defined bipotent or multipotent intermediates. Lineage commitment is thus conceptualized as a fluid and continuous process, in which unilineage progenitors arise directly from low-primed, undifferentiated HSPCs (4). This model emphasizes the plasticity and transcriptional heterogeneity present in early hematopoietic populations and suggests the absence of strict boundaries between stem cells and their committed progeny (5).

B cells play multifaceted roles in the immune system, with antibody production and the positive regulation of adaptive responses representing their most prominent functions (6). Upon antigen recognition, B cells undergo three critical processes: proliferation, immunoglobulin class switch recombination (CSR), and differentiation into plasma cells—all essential for effective antibody production (7). Additionally, B cells can function as antigen-presenting cells, secrete various cytokines, and exert direct cytotoxic effects through Fas ligand (FasL) expression, including against tumor cells (6). However, B cells are also involved in the negative regulation of immunity. They can suppress innate immune responses by secreting IL-10, inhibit T cell proliferation and function via IL-35, and induce CD4+ T cell anergy and apoptosis through the release of TGF-β, among other mechanisms (7).

Although B cell development is regulated by tightly controlled physiological mechanisms, it remains susceptible to genetic disturbances—including mutations and chromosomal translocations—as well as environmental stressors that disrupt homeostasis. Dysregulated B cell development, such as excessive plasma cell proliferation or aberrant autoantibody production, has been implicated in autoimmune diseases, chronic inflammation, cancer progression, and resistance to immunotherapy (8).

## 2 B cell lineages

B cells and the antibodies they produce are central components of humoral immunity (5). They are primarily identified by the presence of the B cell receptor (BCR) on their surface, which binds to specific antigens and initiates a cascade of intracellular signaling events. In addition to their antigen-specific receptors, B cells express major histocompatibility complex class II (MHC II) molecules and possess the full complement of machinery required for antigen uptake, processing, and presentation. These features enable B cells to function as professional antigen-presenting cells (APCs), alongside dendritic cells, monocytes, and macrophages (9).

While B cells also express MHC class I molecules—similar to most nucleated cells—this is not exclusive to them and does not define their role as APCs. Based on developmental origin and function, two major populations of naïve mature B cells have been described: B1 and B2 cells.

### 2.1 B1 cells

B1 cells are long-lived lymphocytes that originate in the fetal liver and bone marrow. They predominantly reside in the pleural and peritoneal cavities, where they are capable of spontaneously differentiating into plasma cells in a T cell-independent manner. These plasma cells secrete natural antibodies that are typically polyreactive, exhibit limited junctional diversity, undergo minimal somatic hypermutation, and display low antigen affinity (10). Nevertheless, B1 cells can also be found in the spleen and other secondary lymphoid organs (11). B1 cells, defined by the surface marker profile CD11b^+^CD21^lo^CD23^lo^CD19^hi^IgM^hi^, are further subdivided into B1a and B1b subsets based on the expression of Ly-1 (CD5), which is present in B1a cells but absent in B1b cells (12). However, CD5 is not an exclusive marker of B1 cells, as it is also expressed by BCR-activated B1b cells, conventional B2 cells, anergic B cells, and B10 regulatory B cells (13). CD11b expression is characteristic of B1a and B1b cells residing in serosal cavities but is downregulated once these cells migrate to other tissues, thereby serving as a distinguishing marker between B1 and conventional B cells (14).

The development of B1 and B2 cells is proposed to occur in three waves for B1 cells and two for B2 cells. The first wave is independent of hematopoietic stem cells (HSC) and takes place around embryonic day 9 in the yolk sac, producing only B1 cells. The second wave occurs during the fetal development, where HSCs in the fetal liver give rise to both B1 and B2 cells. The third wave arises in the adult bone marrow and predominantly generates B2 cells (15). The fetal development of B1a cells is regulated by differential expression of the *Lin28b* and *Let-7* genes, where the first encodes a protein that downregulates the translation and maturation of miRNA Let-7. *Lin28b* encodes a protein that inhibits the maturation of Let-7 microRNA, which in turn targets the transcription factor Arid3a. Higher levels of Arid3a reduce BCR signaling, thereby facilitating the selection of autoreactive BCRs (16). Additionally, the basic helix-loop-helix family member e41 (Bhlhe41), expressed in transitional B1a cells in the neonatal spleen, plays a critical role in regulating their proliferation and survival. This is achieved through the upregulation of the IL-5 receptor α-chain, whose signaling is essential for the self-renewal of B1a cells (17).

B1 cells begin migrating from the fetal liver to body cavities in an immature state, likely as transitional B1 cells (CD93^+^IgM^+^CD23±), which are detected in the spleen during the first two weeks of life. In subsequent weeks, their numbers decline, and B2 cells begin to predominate in the adult spleen. Concurrently, B1 cells appear in serosal cavities, suggesting that transitional B1 cells originate in the spleen and likely enter the peritoneal cavity via the omentum. The omentum contains “milky spots,” aggregates of leukocytes resembling secondary lymphoid tissues. The maintenance of B1 cells within body cavities depends on CXCL13 production, which originates from milky spots and fat-associated lymphoid clusters in the mediastinum, pericardium, and mesentery. The integrins CD11b and CD9 mediate B1 cell adhesion to the visceral and parietal mesothelium of serous membranes. Upon activation of toll-like receptors (TLRs) on B1 cells, these integrins are downregulated, resulting in cell detachment from the extracellular matrix and migration to other anatomical compartments (18).

In contrast to B2 cells, which require recognition of foreign antigens and often depend on CD4^+^ T cell assistance for activation and antibody production, B1 cells can spontaneously differentiate into natural antibody-producing cells (19). B1a cells are the primary source of natural IgM and IgG3 antibodies, which are generated without prior antigenic stimulation and are characterized by polyreactivity and low affinity for pathogens (19). B1 cells recognize T cell-independent (TI) antigens, particularly those with repetitive epitopes such as polysaccharides and phospholipids. As such, they serve as an early defense barrier against pathogenic infections, especially at mucosal sites in the respiratory and gastrointestinal tracts (20). B1a cells and their natural antibodies provide innate protection in naïve hosts, whereas B1b cells are primarily responsible for long-term adaptive antibody responses to TI type 2 antigens, including pneumococcal polysaccharides (PPS) and α1,3-dextran (10). Immunization with TI-2 antigens can induce B1 cells to coexpress the IgM^+^CD27^+^ memory phenotype along with sustained expression of the memory marker CD80, suggesting that they may constitute an additional population of memory B cells (21).

The production of B1 cells ceases shortly after birth, and their long-term maintenance is sustained through self-renewal (22). B1 cells have been implicated in the pathogenesis of autoimmune diseases via several mechanisms, including the production of autoantibodies, antigen presentation, activation of autoreactive CD4^+^ T cells, promotion of Th1 and Th17 cell differentiation, inhibition of regulatory T cell (Treg) development, and cytokine secretion. Notably, increased frequencies of B1 cells have been observed in patients with rheumatoid arthritis (RA), Sjögren’s syndrome, and systemic lupus erythematosus (SLE) (23).

Rheumatoid arthritis (RA) is a chronic systemic inflammatory disorder characterized by synovial fibroblast hyperplasia and varying degrees of bone and cartilage erosion, leading to joint pain, swelling, and reduced mobility (24). Elevated levels of B cell activation markers—such as β2-microglobulin and B cell-activating factor (BAFF) of the tumor necrosis factor family—have been detected in the serum of patients with early-stage RA, suggesting early abnormal B cell activation (25). In this context, B1a cells are known to produce autoantibodies including rheumatoid factor (RF) and anti-citrullinated protein antibodies (ACPAs) (26). RF encompasses antibodies that recognize the Fc region of IgG across various isotypes and affinities and is typically the first biomarker detected in RA (27). However, RF can also be found in individuals with non-rheumatic conditions such as infections or chronic diseases, where it is usually composed of low-affinity, polyreactive IgM antibodies produced by B1a cells. The presence of RF-positive B cells alongside non-autoimmune IgG in healthy individuals implies the existence of tolerance mechanisms. While low-affinity RF may play a role in immune responses to pathogens, high-affinity RF is generally associated with more severe and persistent RA. In combination with ACPAs, RF serves as a central biomarker in the diagnosis of RA (28).

Chronic inflammatory conditions, particularly autoimmune disorders, are associated with an elevated risk of cancer development. The link between B cell hyperactivity and dysregulated cellular immunity contributes to persistent inflammation, which facilitates tumorigenesis in vulnerable tissues (29). Elevated levels of circulating immune complexes (CICs) have been correlated with poor prognosis in patients with breast, genitourinary, head, and neck malignancies (30). The deposition of CICs in the stromal tissue can trigger inflammatory responses by activating the complement cascade and engaging Fc receptors on the surface of leukocytes, further amplifying local immune activation (31).

Evidence indicates that premalignant progression is significantly impaired in the absence of B cells, characterized by reduced recruitment of leukocytes from peripheral blood, failure of the vasculature to initiate angiogenesis, and an inability of hyperproliferative keratinocytes to promote tissue expansion toward a carcinoma *in situ* state. These findings suggest that peripheral B cell activation constitutes an early and essential event in the progression from premalignant lesions to invasive disease (29).

Conversely, the protective role of natural autoantibodies produced by B1 cells has been well documented in several pathological conditions. In atherosclerosis, monoclonal IgM autoantibodies neutralize oxidized low-density lipoproteins (LDL), thereby preventing their pathological uptake by macrophages and reducing inflammation, lesion expansion, and disease progression (32). Additionally, natural IgG enhances the phagocytic clearance of harmful protein aggregates, such as α-synuclein in Parkinson’s disease and β-amyloid in Alzheimer’s disease (33). Natural IgM also confers protection against malignant cells by binding to glycolipids and carbohydrate moieties that are altered during oncogenic transformation (34). Notably, elevated levels of these antibodies have been inversely correlated with tumor development and progression across various cancer types (35). Furthermore, natural IgM targeting double-stranded DNA (dsDNA) is associated with protection against lupus nephritis in patients with systemic lupus erythematosus (SLE) (36).

### 2.2 B2 cells

B2 cells develop in the bone marrow from multipotent hematopoietic stem cells (HSCs) and constitute the majority of the B cell population circulating in adult humans (37). Follicular (FO) B cells undergo further differentiation within germinal centers (GCs) upon activation by CD4^+^ T cells and play a central role in the T cell-dependent B cell response of the adaptive immune system. Ultimately, they differentiate into plasma cells capable of producing high-affinity antibodies. In contrast, marginal zone (MZ) B cells express B cell receptors (BCRs) that preferentially recognize blood-borne pathogens, particularly bacterial cell wall components. Upon stimulation through Toll-like receptors (TLRs), immature B cells are recruited to the marginal zone and differentiate into IgM-secreting plasma cells, serving as a rapid, first-line defense against pathogens that access the spleen (39). The development and functional specialization of these B2 cell subsets will be further detailed in the following section.

#### 2.2.1 Regulatory B cells

Regulatory B cells (Bregs) are a minor subpopulation of B2 cells, comprising less than 1% of human peripheral blood mononuclear cells, and are known for their immunomodulatory functions (9). They act as negative regulators of the immune system by preventing potentially harmful autoimmune processes that lead to uncontrolled inflammation (40). Bregs exert their effects either by secreting immunoregulatory cytokines—such as IL-10, TGF-β, and IL-35—or through cell-to-cell contact mechanisms involving granzyme B, programmed death-ligand 1 (PD-L1), CD39, CD73, FasL, and glucocorticoid-induced TNFR-related ligand (GITRL). IL-10 has potent anti-inflammatory properties and protects against tissue damage in allergic diseases, autoimmune disorders, organ transplantation, and tumor tolerance (41). IL-10–producing B cells can also inhibit effector B cell proliferation (42). In parallel, TGF-β promotes the differentiation of CD4^+^ T cells into regulatory T (Treg) cells and limits their proliferation and differentiation into Th1 and Th17 cells (43). Additionally, Bregs can induce apoptosis in CD4^+^ T cells and anergy in CD8^+^ effector T cells, thereby contributing to tissue remodeling and wound healing processes (44). Finally, IL-35 attenuates the pathogenic effects of Th1 and Th17 cells in autoimmune diseases (38).

Their suppressive functions affect various immune cells, including T cells, DCs, and monocytes (27). B cells that mediate regulatory functions exclusively through IL-10 are referred to as B10 cells. These are characterized by a CD1d^+^CD5^+^ immunophenotype and represent the most abundant Breg population in the spleen (28). Bregs can originate from immature and mature B cell subsets—including MZ B cells, transitional 2-marginal zone precursors, B10 cells, B1a cells, plasmablasts, and plasma cells—upon antigen recognition and exposure to stimuli such as TLR ligands, CD40 activation (the most well-characterized induction signal), CpG oligodeoxynucleotides, IL-2, IL-6, and IFN-α. The transcription factors IRF8 and IRF4 regulate IL-10 production by controlling the expression of immunosuppressive gene loci, including IL-10 and IL-35 subunits (EBI3 and IL12A, respectively) in Bregs (38). These findings support the notion that Breg differentiation is driven less by a unique lineage-defining factor and more by the immunological environment in which the B cell resides (45).

Multiple transcription factors have been implicated in the dynamic regulation of Breg differentiation and function, depending on the immunological context. For instance, low expression levels of IRF4 and PRDM1 (Blimp-1) favor the expansion of IL-10–producing Bregs, with both factors acting as negative regulators of the regulatory phenotype (46). Conversely, recent studies have identified STING as a cytosolic sensor that, when activated in B cells, induces an immunosuppressive profile characterized by increased IL-10 and PD-L1 expression, thereby compromising NK cell function in the tumor microenvironment. Although the specific transcriptional mediators remain unclear, pathways involving IRF3 or NF-κB are likely involved (47). Furthermore, single-cell transcriptomic analyses have identified a set of 19 genes commonly expressed in Bregs, including *Fcrl5*, *Zbtb20*, *Ccdc28b*, *Cd9*, and *Ptpn22*. Among these, ATF3 stands out as a transcription factor whose expression correlates with key regulatory genes such as *Il10* and *Pdcd1lg2*, suggesting its potential role as a positive modulator of the Breg phenotype (48). These data reinforce the concept that Breg identity results from an integrated regulatory network, rather than a single master transcriptional regulator, with its activation tailored to specific microenvironmental signals.

The differentiation of Bregs is strongly influenced by inflammatory stimuli, which serve as critical enhancers of their immunosuppressive phenotype. Cytokines such as BAFF and APRIL, frequently elevated in chronic inflammatory conditions, not only promote B cell survival but also induce the expression of regulatory mediators such as IL-10, thereby facilitating the development of a functional Breg profile (49). *In vitro* studies have shown that combined stimulation with CpG (a TLR9 agonist), CD40L, and IL-21 is particularly effective at inducing B cells with suppressive capabilities, highlighting the synergistic role of TLR engagement and costimulatory signals in programming the regulatory phenotype (50). These findings suggest that, although inflammation is not the sole requirement, it is a decisive factor in promoting Breg differentiation, particularly in immunologically active environments such as those observed in autoimmune and allergic diseases.

Regulatory B cells (Bregs) exhibit a dual role across various pathologies, modulating immune responses in ways that can be either beneficial or detrimental depending on the disease context. In cancer, numerous studies have demonstrated that Bregs facilitate tumor immune evasion by secreting IL-10 and TGF-β and expressing PD-L1, thereby suppressing cytotoxic T and NK cell activity and contributing to tumor progression. These effects have been documented in gastric, bladder, and other solid tumors, where increased Breg abundance correlates with poor prognosis and reduced responsiveness to immunotherapies (47, 51–54). Conversely, in the context of organ transplantation, Bregs promote tolerance by suppressing immune responses that could otherwise lead to graft rejection. Notably, Granzyme B–producing Bregs have been identified in tolerant kidney transplant recipients, and TIM-1 has been proposed as a relevant marker for their expansion and clinical monitoring (55, 56). These findings position Bregs as central regulators of immune balance, capable of either promoting or mitigating disease progression depending on the immunological setting.

## 3 B2 cell development

B2 cells originate from multipotent HSCs, first appearing within the para-aortic splanchnopleure of the embryo. They subsequently colonize the fetal liver around the seventh week of gestation and, finally by the middle of the second trimester, are permanently established in the bone marrow, where B cell generation continues throughout life (57). In general, B cell development and differentiation require the coordinated action of multiple cytokines, transcription factors (TFs), and cell-surface molecules that orchestrate gene expression programs essential for lineage specification and maturation (58).

### 3.1 Structure and signaling of the BCR

The B cell receptor (BCR) is a transmembrane immunoglobulin complex located on the surface of B cells. It plays a pivotal role in immune responses, influencing processes such as cell proliferation, adhesion, differentiation, survival, cytoskeletal remodeling, and apoptosis (59). A mature BCR consists of a membrane-bound immunoglobulin (mIg) formed by two immunoglobulin heavy (IgH) chains, encoded by the IgH locus on chromosome 14, and two immunoglobulin light (IgL) chains, encoded by either the Igκ locus (chromosome 2) or the Igλ locus (chromosome 22) (60). The IgH chains are categorized into five major isotypes—Igμ, Igα, Igγ, Igδ, and Igϵ—each comprising four (Igα, Igγ, Igδ) or five (Igμ, Igϵ) constant domains. The IgL chains, including Igκ and Igλ, contain only two constant domains. The N-terminal regions of both chains, known as variable (V) regions, exhibit extensive sequence variability and are responsible for antigen binding. In contrast, the C-terminal regions are more conserved among individuals of the same species and are thus referred to as constant (C) regions. The variable region of the IgH chain is encoded by the recombination of V (variable), D (diversity), and J (joining) gene segments, whereas the variable region of the IgL chain is encoded by V and J segments only, lacking D segments (61).

In addition to mIg, the BCR includes a signaling subunit composed of a disulfide-linked heterodimer of Igα and Igβ proteins (CD79A and CD79B, respectively) (7). Each subunit contains a cytoplasmic immunoreceptor tyrosine-based activation motif (ITAM), characterized by two conserved tyrosine residues within a consensus sequence, which serves as a docking site for SH2 domain-containing effector proteins (62). These subunits are essential for BCR assembly, stabilization, and the transport of IgM to the cell surface. They also enhance BCR surface expression by regulating its glycosylation (63).

The Igα/Igβ heterodimer assembles via interactions between their extracellular domains (ECDs), membrane-proximal connecting peptide regions, and four transmembrane (TM) helices. The fragment crystallizable (Fc) region of the mIg stabilizes the complex by interacting with the ECDs of Igα and Igβ. The TM helices form a compact bundle stabilized by conserved hydrophobic and polar interactions. The intracellular domains (ICDs) of these subunits remain unresolved in structural analyses due to their intrinsic flexibility (58). While the BCR is the primary receptor guiding B cell differentiation, it integrates additional signals from co-receptors such as CD19, CD21, CD22, CD72, and FcγRIIB (64).

CD19 is a B cell-specific co-receptor that amplifies BCR-mediated signaling (21). It is typically associated with a membrane complex composed of CD21, CD81, and Leu-13. As a membrane adaptor, CD19 recruits critical signaling molecules, including Vav, phosphoinositide 3-kinase (PI3K), and Lyn, and contributes to the activation of downstream pathways such as phospholipase C-γ2 (PLC-γ2) and the mitogen-activated protein kinase (MAPK) cascade. It serves two major roles: first, as an adaptor protein facilitating the recruitment of cytoplasmic signaling molecules to the membrane, and second, as a signaling subunit for the CD19/CD21 complex upon co-ligation with the BCR (65). Therefore, the CD19–CD21 complex modulates the threshold for B cell activation (66). In humans, CD19 mutations result in severe immunodeficiency, whereas CD19 overexpression in mice leads to defects in early B cell development, hyperresponsiveness to stimulation, and autoimmune-like phenotypes (67).

Upon antigen engagement, the BCR initiates two critical processes: transmembrane signaling and antigen internalization. The former triggers a cascade of protein tyrosine phosphorylation and intracellular calcium influx, leading to the upregulation of surface molecules required for B–T cell collaboration and entry into the cell cycle. Meanwhile, antigen internalization facilitates proteolytic processing and loading of antigen-derived peptides onto MHC class II molecules for subsequent presentation to CD4^+^ T cells (66).

**Figure 2:**
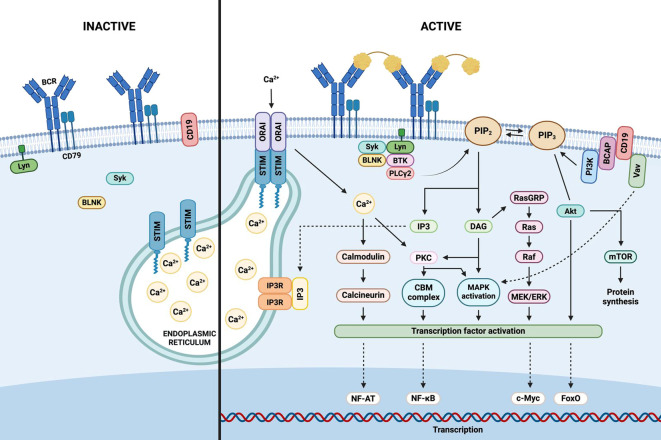
B cell receptor signaling. Antigenic stimulation of the extracellular domain of the B cell receptor (BCR) triggers the assembly of a signalosome, comprising the BCR complex, protein tyrosine kinases, adaptors, and effector molecules. Activation of PI3K (phosphoinositide 3-kinase) catalyzes the conversion of PIP2 (phosphatidylinositol 4,5-bisphosphate) to PIP3 (phosphatidylinositol 3,4,5-trisphosphate), and also activates PLC-γ2 (phospholipase C-γ2), generating IP3 (inositol 1,4,5-trisphosphate) and DAG (diacylglycerol) from PIP2. IP3 binds to its receptor (IP3R) on the endoplasmic reticulum (ER), prompting calcium release. Calcium depletion is sensed by STIM (stromal interaction molecule), which oligomerizes and translocates to ER–plasma membrane junctions to activate Orai channels, resulting in calcium influx from the extracellular space. Calcium, IP3, and DAG initiate downstream signaling cascades including PI3K-AKT, NF-κB, and Ras-MAPK pathways, ultimately activating transcription factors and nuclear gene expression that promote B cell survival and proliferation.

The BCR elicits two distinct signaling modes: a continuous, low-intensity “tonic” signal—mediated by the PI3K/AKT/mTOR axis and independent of antigen binding—and an “activated” signal triggered by antigen engagement, which recruits downstream cascades promoting B cell proliferation, survival, and differentiation (68). Antigenic stimulation induces BCR oligomerization and translocation into cholesterol-enriched lipid rafts, where ITAMs are phosphorylated by Src-family kinases such as Lyn, Fyn, and Blk (69). This leads to the activation of three major signaling pathways: nuclear factor kappa-light-chain-enhancer of activated B cells (NF-κB), Ras–MAPK, and PI3K–AKT, as illustrated in **Figure 2** (70).

#### 3.1.1 NF-κB pathway

Upon antigen binding to the BCR, Src-family tyrosine kinases Lyn, Fyn, and Blk phosphorylate the ITAMs located in the cytoplasmic tails of Igα/Igβ. The spleen tyrosine kinase (Syk) subsequently binds to the phosphorylated ITAMs and becomes activated, leading to the tyrosine phosphorylation of the B cell linker protein (BLNK). Concurrently, these kinases also phosphorylate CD19 and Bruton’s tyrosine kinase (BTK). Activated BTK, in turn, phosphorylates and activates phospholipase C-γ2 (PLC-γ2), which hydrolyzes phosphatidylinositol (4, 5)-bisphosphate (PIP2) into two second messengers: diacylglycerol (DAG) and inositol 1,4,5-triphosphate (IP3) (71).

IP3 induces the release of intracellular calcium stores, while DAG activates protein kinase C (PKC). Activated PKC then phosphorylates the CARD11–BCL10–MALT1 (CBM) signaling complex (72). This event triggers the activation of the inhibitor of κB kinase (IKK) complex, which subsequently phosphorylates IκB proteins, leading to their degradation and the release of free NF-κB dimers. These NF-κB dimers translocate to the nucleus, where they regulate the transcription of genes involved in B cell proliferation, survival, and differentiation (70).

#### 3.1.2 Ras-MAPK pathway

The Ras–MAPK pathway plays a critical role in B cell proliferation and survival (73). DAG, produced by PLC-γ2 activity, binds to and activates Ras guanyl nucleotide-releasing protein (RasGRP), which, in turn, activates the membrane-bound molecular switch protein Ras by catalyzing the exchange of GDP for GTP. The active Ras–GTP complex recruits Raf kinases to the plasma membrane, where they are activated via enzymatic transphosphorylation and dimerization (74).

Activated Raf phosphorylates and activates the mitogen-activated protein kinase kinases (MAPKKs) MEK1 and MEK2. These MEK kinases subsequently phosphorylate and activate extracellular signal-regulated kinases 1 and 2 (ERK1/2). Once activated, ERK1/2 translocate into the nucleus, where they phosphorylate various transcription factors (TFs), including c-Myc, Myb, Elk, and others, ultimately regulating gene expression related to cell growth and differentiation (75).

#### 3.1.3 PI3K-AKT pathway

Phosphoinositide 3-kinase (PI3K) is recruited to the plasma membrane via CD19. Upon activation, PI3K phosphorylates phosphatidylinositol (4, 5)-bisphosphate (PIP2), converting it into phosphatidylinositol (3–5)-trisphosphate (PIP3). PIP3 then serves as a docking site for downstream signaling molecules, including BTK. This kinase is subsequently activated by phosphorylation via Src family kinases, leading to the activation of PLC-γ2, which hydrolyzes PIP2 to generate inositol 1,4,5-trisphosphate (IP3) and diacylglycerol (DAG) (76).

The IP3 receptor (IP3R), a calcium channel located in the endoplasmic reticulum, responds to IP3 binding by releasing calcium into the cytosol. This calcium influx activates the phosphatase calcineurin, which dephosphorylates and activates the nuclear factor of activated T cells (NFAT). Additionally, the combination of increased cytosolic calcium and DAG activates PKC, which phosphorylates the adaptor protein CARD11, thereby initiating the CARD11–BCL10–MALT1 (CBM) signaling complex that also culminates in the activation of NF-κB (77).

BCR signal transduction is tightly regulated by a network of inhibitory receptors, including CD22, CD72, FCγRIIB, and SIGLEC10. Negative regulation is also mediated by phosphatases such as SHP1, protein tyrosine phosphatase non-receptor type 22 (PTPN22), SHIP1, and phosphatase and tensin homolog (PTEN) (78). Specifically, SHP1 and PTPN22 dephosphorylate critical signaling components such as CD79A, CD79B, Src family kinases (SFKs), and BLNK, whereas PTEN and SHIP1 dephosphorylate PIP3, thereby attenuating PI3K signaling (77).

Activated B cell-like diffuse large B cell lymphoma (ABC-DLBCL), a subtype of B cell non-Hodgkin lymphoma (B-NHL), is characterized by chronic active BCR signaling, which leads to constitutive activation of the NF-κB pathway (79). This aberrant signaling is driven by multiple mechanisms, including gain-of-function mutations in CARD11, loss-of-function mutations in A20—a negative regulator of NF-κB—and mutations within ITAMs that impair BCR endocytosis, thereby enhancing surface BCR expression (78). Similarly, mantle cell lymphoma (MCL) is characterized by constitutive phosphorylation of BTK and PLC-γ2, underscoring the importance of sustained BCR signaling in promoting malignant B cell survival and proliferation (80).

Ibrutinib is a covalent BTK inhibitor that irreversibly binds to its active site, thereby blocking BTK’s enzymatic activity and downstream signaling. This mechanism has proven critical in the treatment of various B cell non-Hodgkin lymphomas (B-NHL) (81). However, resistance to ibrutinib has been observed, with resistant cells often exhibiting constitutive activation of the AKT pathway. This can arise from point mutations in the BTK gene that enhance BTK-mediated signaling and activate the AKT cascade, or from loss-of-function mutations in PTEN, resulting in persistent phosphorylation and activation of BCR signaling components. Therefore, therapeutic strategies combining BTK inhibitors with agents targeting the PI3K/AKT pathway are being explored for the treatment of MCL (80).

Chronic lymphocytic leukemia (CLL), the most prevalent lymphoproliferative disorder, is characterized by autonomous and antigen-independent BCR signaling. This signaling persists despite impaired glycosylation and defective folding of the IgM and Igα chains, and is associated with the retention of surface BCR expression (82). Schmid and Hobeika, using Eμ-TCL1 mouse models, demonstrated that induced loss of the Igα subunit leads to near-complete elimination of neoplastic cells, underscoring the indispensable role of BCR signaling in CLL cell survival (63).

The CD3ζ-chain-associated protein of 70 kDa (ZAP-70), originally identified in T cells, is also aberrantly expressed in CLL B cells and serves as a prognostic biomarker. ZAP-70 enhances BCR signaling independently of the phosphorylation status of its activating tyrosines (83). This tonic BCR signaling driven by ZAP-70 promotes transcriptional upregulation of oncogenic targets such as MYC and chemokines CCL3 and CCL4, which recruit T cells into proliferation centers, ultimately supporting CLL cell survival and expansion (84).

### 3.2 Development and maturation

**Figure 3:**
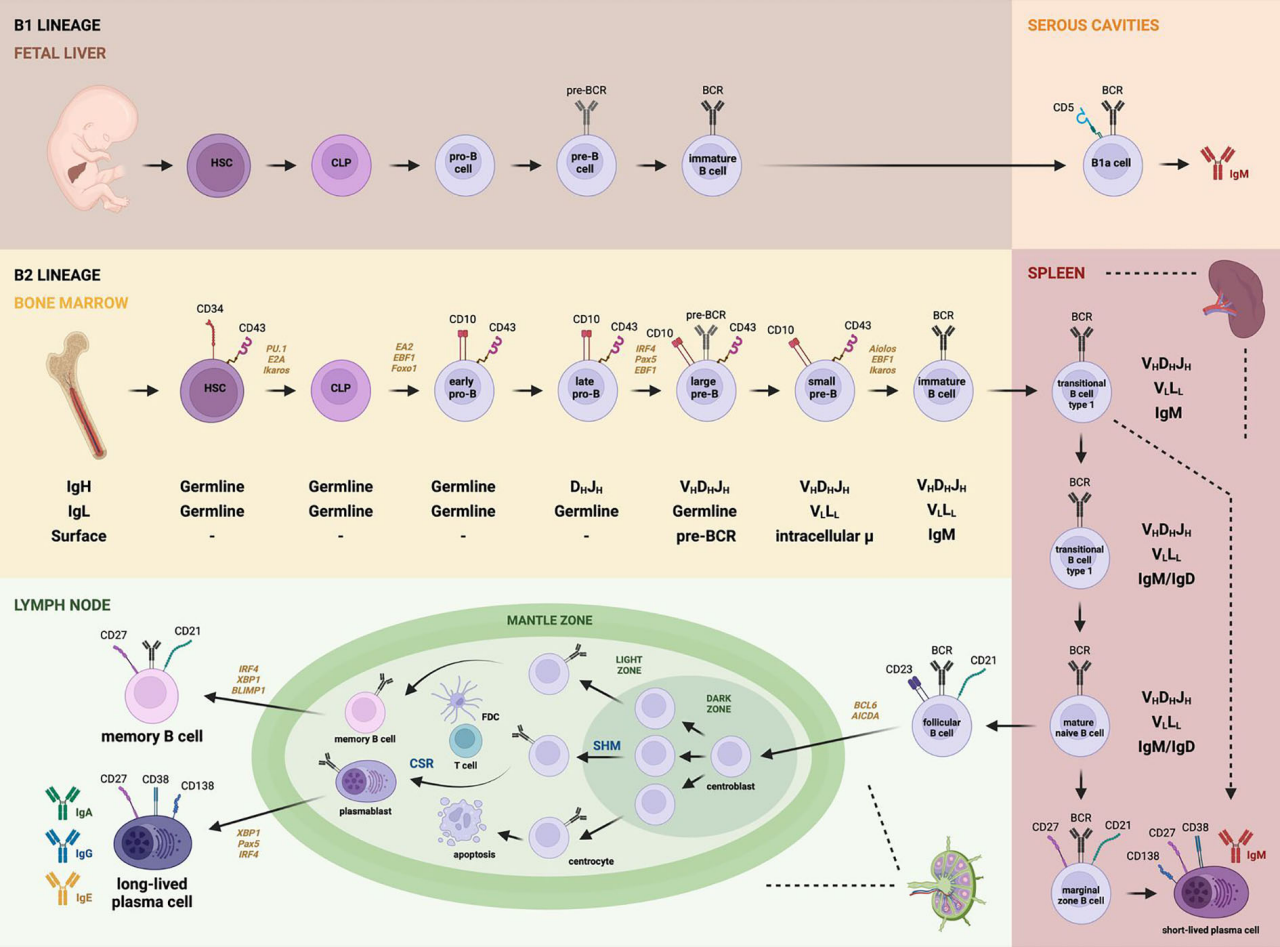
B cell development. B1 lineage cells originate in the fetal liver and follow a developmental pathway from HSCs to CLPs, progressing through pro-B cells, pre-B cells, and immature B cells, before migrating to serous cavities where they differentiate into B1a or B1b cells capable of secreting natural IgM antibodies. In contrast, the B2 lineage originates in the bone marrow and follows a similar progression—beginning with HSCs and CLPs, through early and late pro-B cells, large and small pre-B cells, and immature B cells. Upon migration to secondary lymphoid organs (SLOs) such as the spleen, these cells become transitional B cells. Transitional cells may differentiate into marginal zone B cells in the spleen (producing short-lived plasma cells) or migrate to lymph nodes, giving rise to follicular B cells. In the germinal centers, follicular B cells become centroblasts, undergo somatic hypermutation (SHM) and class switch recombination (CSR), and interact with T follicular helper cells and dendritic cells. This leads to immunoglobulin diversification and maturation into memory B cells and plasmablasts, which ultimately give rise to memory B cells and long-lived plasma cells secreting IgA, IgG, or IgE. Notably, pre-BCR expression begins at the large pre-B cell stage, while the mature BCR is expressed from the immature B cell stage onward. This figure also highlights the dynamics of V(D)J recombination, expression of specific CD markers, and transcription factors relevant to each differentiation stage.

As illustrated in **Figure 3**, B cell development and maturation encompass two distinct but sequential processes: antigen-independent precursor B cell differentiation, which occurs in the bone marrow, and antigen-dependent B cell maturation, which takes place in secondary lymphoid organs (SLOs). The primary objective of precursor B cell differentiation is the generation of a functional immunoglobulin (Ig) receptor through the ordered V(D)J recombination of the gene segments encoding the Ig heavy (IgH) and light (IgL) chains. Following the successful generation of a functional IgH protein and the expression of the pre-B cell receptor, pre-B cells undergo multiple rounds of proliferation before initiating IgL chain rearrangement (85).

The early, bone marrow-dependent stages of B cell development are tightly linked to the stepwise recombination of immunoglobulin gene segments. Specifically, recombination of the VH, DH, and JH segments for the immunoglobulin heavy (IgH) chain, along with the VL–JL segments for the light (IgL) chain, collectively generates a highly diverse B cell repertoire capable of recognizing over 5 × 10¹³ distinct antigens.

In the pro-B cell stage, the initial recombination involves the D–J segments of the IgH locus, followed by the joining of an upstream V region to the rearranged DJ segment. Successful completion of this process allows progression to the pre-B cell stage, which is marked by the expression of a functional μ heavy chain (μ-HC) and the formation of the pre-B cell receptor. At this stage, the cell undergoes one or two rounds of division and initiates rearrangement of the κ and λ IgL chain genes. Following successful light chain rearrangement and surface expression of a complete IgM molecule, the cell enters the immature B cell stage. These immature B cells then exit the bone marrow and migrate to secondary lymphoid organs (SLOs), where they complete their maturation and become capable of participating in antigen-dependent immune responses (62).

#### 3.2.1 Antigen-independent precursor B cell differentiation

The expression of CD19 by lymphoid progenitors marks the commitment to the B cell lineage and initiates a series of immunoglobulin chain rearrangements essential for B cell development (11). The main stages of early B cell differentiation include the pro-B, pre-B, and immature B cell stages, each defined by specific surface markers and sequential recombination events at the immunoglobulin gene loci. During the early pro-B cell stage, B cell progenitors begin recombining the D and J segments of the IgH locus. This is followed by the joining of a V segment to the D-J segment, thus completing the late pro-B cell stage (37).

Following successful IgH chain rearrangement, pro-B cells initiate expression of the pre-B cell receptor (pre-BCR), which consists of the rearranged μ heavy chain (μ-HC), surrogate light chain components (V-preB or CD179A and λ5 or CD179B), and the signal-transducing subunits Igα (CD79A) and Igβ (CD79B) (86). The pre-BCR serves two critical functions: first, it mediates allelic exclusion by halting further IgH rearrangement to prevent the expression of two distinct heavy chains in a single cell; second, it triggers the initiation of IgL chain gene rearrangement (62). Upon pre-BCR expression, the cell enters the pre-B stage, wherein the VL and JL gene segments recombine to generate the light chains, kappa (κ) or lambda (λ) (37).

Pre-BCR signaling also results in increased intracellular calcium levels and acts as a proliferative stimulus, enabling clonal expansion of pre-B cells (87). This signaling is essential for B cell development, as it downregulates RAG1/2 protein expression and drives the proliferation of large pre-B cells. Subsequently, the expression of the cytoplasmic adaptor protein SLP-BLNK promotes the transition to small pre-B cells by inhibiting the PI3K/Akt pathway and inducing FOXO1, which leads to surface expression of IgM as a complete BCR on immature B cells (88).

Once IgL chains are successfully rearranged, B cell precursors reach the final stage of antigen-independent development. Immature B cells express surface IgM and are subject to central tolerance mechanisms. Cells exhibiting high-affinity binding to self-antigens undergo clonal deletion or receptor editing via additional IgL rearrangements (89). Immature B cells that exit the bone marrow differentiate into transitional B cells, which co-express IgM and IgD upon entry into the spleen or lymph nodes (90). IgD expression raises the activation threshold, allowing the B cell to remain tolerant to self-antigens while preserving the ability to respond to foreign antigens (37).

In summary, the surface of B cells is composed of membrane-bound immunoglobulins, complement receptors, Fc receptors, and approximately ten B cell-specific surface molecules classified as clusters of differentiation (CD) (91). Human B-lineage cells undergo a well-defined progression of CD marker expression: early B cells (CD34^+^CD19^-^CD10^+^), pro-B cells (CD34^+^CD19^+^CD10^+^), pre-BI cells (CD34^-^CD19^+^CD10^+^), large pre-BII cells (CD34^-^CD19^+^CD10^+^), small pre-BII cells (CD34^-^CD19^+^CD10^+^), immature B cells (CD34^-^CD19^+^CD10^+^), and mature B cells (CD34^-^CD19^+^CD10^-^) (92).

#### 3.2.2 Antigen-dependent B cell maturation

Transitional B cells represent the final stage of development before differentiation into the mature pre-immune B cell pool. Based on the expression of specific surface markers, transitional B cells are further categorized into two subsets: T1 and T2. T1 B cells, characterized by the phenotype IgM^hi^IgD^–^CD21^–^CD23^–^, can be found in the bone marrow, blood, and spleen, but not in lymph nodes. In contrast, T2 B cells are exclusively found in the spleen, express IgM^hi^IgD^+^CD21^+^CD23^+^, and give rise either to circulating B cells that participate in germinal center (GC) formation or to non-circulating marginal zone (MZ) B cells (9, 93).

Within the spleen, transitional B cells differentiate into several subtypes, including MZ B cells, regulatory B cells (Bregs), follicular (FO) B cells, activated B cells, GC B cells, plasma cells (both short- and long-lived), and memory B cells (12). MZ B cells rapidly differentiate into IgM-secreting short-lived plasma cells, serving as a first line of defense against blood-borne pathogens (39). FO B cell activation is triggered when antigens enter B cell follicles in SLOs, whereupon antigen recognition and processing leads to presentation via MHC class II to CD4^+^ T cells. These T cells, in turn, provide costimulatory signals, most notably through CD40 ligand (CD40L) binding to CD40 on B cells. The strength of BCR signaling, modulated by additional inputs such as Notch2, dictates B cell fate: weak signals favor MZ B cell differentiation, whereas stronger BCR signals promote FO B cell development (8).

Upon activation, FO B cells can differentiate into rapidly dividing blasts that initiate GC formation (94). The GC is anatomically and functionally divided into two zones: the dark zone, populated by proliferating centroblasts undergoing somatic hypermutation (SHM) and class switch recombination (CSR); and the light zone, which contains centrocytes—post-mitotic B cells undergoing selection based on antigen affinity. Follicular dendritic cells (FDCs) and CD4^+^ T cells are enriched in the light zone, facilitating positive selection of high-affinity B cell clones that will become either plasma cells or memory B cells (95). CXCR4 expression is critical for GC B cell positioning in the dark zone, where its ligand CXCL12 is most abundant (96).

The transcription factor T-bet, encoded by *TBX21*, is well known for its role in T helper 1 (Th1) differentiation, but also exerts essential functions in B cells. T-bet promotes class switching to IgG2c, supports B cell commitment to the dark zone, and is upregulated in memory B cells and aged B cells responding to viral infections (97). T-bet-driven gene expression may help retain low-affinity, antigen-experienced B cells within the GC, thus fine-tuning the quality of humoral responses (95).

Centroblasts eventually exit the cell cycle, re-express surface immunoglobulins, and become smaller centrocytes that migrate to the light zone. There, they undergo affinity-based selection, competing for antigen presented as immune complexes by FDCs and interacting with CD4^+^ T cells to determine differentiation into antibody-secreting plasma cells or long-lived memory B cells (96). GC B cells are among the fastest dividing mammalian cells, with cycle times ranging from 6 to 12 hours, eventually displacing FO B cells to the outer mantle zone of the GC (39).

Light zone B cells with lower-affinity BCRs and minimal T cell help express higher levels of the transcriptional repressor Bach2, predisposing them to enter the memory B cell pool, particularly in early GC responses (98). The transition from GC to memory B cells requires cessation of proliferation in the dark zone, migration to the light zone, and entry into a quiescent, survival-favored state (99).

Sphingosine-1-phosphate (S1P), a lysosphingolipid, plays a crucial role in lymphocyte migration from lymphoid organs to the circulation. S1P binds to five G protein-coupled receptors (S1PR1–S1PR5), each with distinct tissue distributions. S1PR1–S1PR3 are broadly expressed, S1PR4 is confined to lymphoid and hematopoietic tissues, and S1PR5 is predominant in the central nervous system (91, 100). S1PR1 promotes B cell egress from SLOs and regulates the positioning of MZ B cells and plasma cells. In contrast, S1PR2 antagonizes CXCR4 and CXCR5 signaling to retain GC B cells, while S1PR4 modulates T cell migration (92).

Following antigen uptake, activated B cells downregulate S1PR1, migrate to the outer follicular regions, and engage with antigen-specific CD4^+^ T cells. Before moving to the T cell zones, B cells first migrate outward—a process mediated by the G protein-coupled receptor EBI2 (GPR183), which is expressed on B, T, NK, dendritic cells, and others, but downregulated in GC B cells. EBI2 plays a pivotal role in positioning cells throughout the immune response (101). One of its ligands, 7α,25-dihydroxycholesterol (7α,25-OHC), is enriched in the inter-follicular and outer follicular regions but absent from the follicle center (102). Downregulation of EBI2, mediated by the transcriptional repressor BCL6, is necessary for B cell migration into follicle centers and GC initiation (103). Conversely, EBI2 overexpression leads to B1a cell expansion, diminished immune responses, oncogene activation, and late-onset lymphoid malignancies, resembling features of chronic lymphocytic leukemia (CLL) (101).

Migration into the T cell zone is regulated by CCR7, which facilitates CD40–CD40L interactions at the T-B border. CD40L (CD154), a transmembrane cytokine expressed by activated CD4^+^ T cells, engages CD40 on B cells to drive T cell-dependent antibody responses. CD40L is essential for GC maintenance, affinity maturation, CSR, and long-lived plasma cell generation. Upon contact with B cells, CD40L is transferred and retained on their surface and within the cytoplasm, enhancing ICAM-1 expression and cellular activation (104). This mechanism supports the maintenance of polyreactive B cells that contribute to immune defense despite their weak autoreactivity (105).

T follicular helper (Tfh) cells, defined by expression of CXCR5, PD-1, BCL6, and cytokines such as IL-21 and IL-4, are central to GC responses. They guide B cell differentiation, maturation, and survival (106). The chemokine CXCL13, also known as BCA-1 or BLC, attracts CXCR5^+^ B and T cells into SLO B cell follicles. Follicular dendritic cells and GC Tfh cells produce CXCL13 to drive follicular organization. At the T-B cell interface, FO B cells facilitate Tfh differentiation, which in turn enhances GC formation and antibody affinity maturation (107). Additionally, CXCL13 from peritoneal macrophages attracts B1 cells, contributing to innate immunity in body cavities (108).

Co-stimulation through CD40L and Tfh-derived IL-4/IL-21 enables activated B cells to migrate with Tfh cells via CXCR5 toward CXCL13-enriched follicle centers, where they establish new GCs (39). Centroblasts expressing high CXCR4 are retained in CXCL12-rich dark zones for proliferation and SHM. Resting lymphocytes expressing S1PR1 respond to S1P gradients to exit SLOs. Recirculating mature FO, MZ, and memory B cells express S1PR1 and S1PR4, but not S1PR2. In contrast, non-recirculating GC B cells and extrafollicular plasma cells express high S1PR2 and S1PR4 levels. S1PR2 inhibits chemokine-induced migration, contributing to the spatial organization and functional segregation of GCs into dark and light zones, allowing cycles of mutation and selection (39).

miR-146a regulates GC B cell responses primarily through modulation of the CD40 signaling pathway. Its loss leads to spontaneous immune activation, indicating its essential regulatory role. While miR-146a is crucial for Treg-mediated immune homeostasis, it appears dispensable for Tfh cell regulation. Dysregulated miR-146a expression has been implicated in autoimmune diseases such as systemic lupus erythematosus (SLE), where its expression negatively correlates with disease progression (109).

In summary, HSCs give rise to common lymphoid progenitors (CLPs, CD34^+^CD10^+^CD19^–^), initiating B cell lineage commitment with D–J gene rearrangement and forming CD34^+^CD10^+^CD22^+^CD19^–^ pre-pro-B cells. V–DJ recombination and CD19 expression define the pro-B cell stage (CD34^+^CD10^+^CD19^+^). Successful μ heavy chain expression leads to pre-BCR formation, transitioning cells into large pre-B cells (CD34^–^CD10^+^CD19^+^), which subsequently cease surrogate light chain expression and undergo κ or λ light chain rearrangement. This culminates in mature BCR expression and the appearance of additional markers such as CD20, CD21, CD22, and CD40 (110, 111).

These mature B cells exit the bone marrow as transitional B cells co-expressing IgM and IgD. Upon entry into SLOs, they differentiate into either MZ B cells, which rapidly generate short-lived plasma cells producing IgM, or FO B cells, which migrate to lymph nodes. FO B cells, upon antigen stimulation and aided by BCL6 and AID, initiate GC reactions, ultimately generating high-affinity memory B cells and long-lived plasma cells capable of secreting IgA, IgG, or IgE (111).

### 3.3 Transcriptional factors in B lymphocytes

Both B and T cell precursors initiate their developmental pathways through a cascade of transcriptional events that activate genes responsible for antigen receptor assembly and signaling during the common lymphoid progenitor (CLP), pre-B, and pro-B stages of B cell differentiation. Precursors from both lineages depend on the Ets family transcription factor PU.1 for their generation and survival, along with the zinc-finger transcription factors Ikaros (Ikzf1) and BCL11A, as well as the basic helix-loop-helix (bHLH) E protein family member E2A (Tcfe2a). Notably, the functions of Ikaros, BCL11A, and E2A are essential for B cell development (61).

At least ten transcription factors regulate the early stages of B cell development, with Ikaros, Aiolos, PU.1, E2A, early B cell factor (EBF), and Pax5 (a paired-box transcription factor exclusively expressed in the B lineage) playing particularly critical roles in promoting B cell lineage commitment and differentiation (22). PU.1 regulates the expression of CD79A, RAG1, and terminal deoxynucleotidyl transferase (TdT), as well as all three immunoglobulin loci: μ, κ, and λ. Ikaros promotes the expression of TdT, λ5, and VpreB. The E2A locus encodes two bHLH transcription factors, E12 and E47, which are alternatively spliced products. E12 primarily promotes the expression of early EBF (which is expressed at all stages of differentiation except in plasma cells) and Pax5, whereas E47 enhances the expression of TdT and RAG1 more effectively (112).

Some members of the Ikaros family, which contain zinc-finger motifs, are involved in the specification of hematopoietic stem cells (HSCs) to the lymphoid lineage. During IgL-chain rearrangement, Aiolos, another Ikaros family member, plays a crucial role in downregulating λ5 expression in pre-B cells. Ikaros and Aiolos form a regulatory network connected by a feedback loop that guides the transition from pro-B to immature B cells (39).

Commitment to the B cell lineage in CLPs is initiated by E12/E47 (E2A) and early B cell factor 1 (EBF1). These factors cooperatively induce the expression of a suite of B lineage-specific genes, including *CD79A, CD79B, CD179A, PAX5*, and *FOXO1* (39). The threshold for initiating B cell development is reached when EBF1 and Pax5 are upregulated *de novo*. These transcription factors are exclusively expressed in B-lineage cells among all hematopoietic types, establishing a self-sustaining regulatory network (61). n pre-pro-B cells, transcription factors associated with the myeloid lineage (e.g., RUNX2, IRF8, BST2, and TCF4) are downregulated as cells progress to the pro-B stage. The transition from pro-B to pre-B cells requires the expression of LEF1 for survival and proliferation (8). Additionally, SOX4 and FOXO1 are essential for activating the recombination-activating genes RAG1 and RAG2, which mediate IgH and IgL gene rearrangements. Pax5 not only initiates transcription of CD19 and BLNK but also represses genes such as NOTCH1 and CSF1R, thereby inhibiting T and myeloid lineage development. Thus, E2A, EBF1, FOXO1, and Pax5 orchestrate the most critical steps of early B cell development, including V(D)J recombination and expression of the pre-BCR components (39).

During the germinal center (GC) reaction, interactions between GC stromal cells and T follicular helper (Tfh) cells allow B cells to undergo somatic hypermutation (SHM) and class-switch recombination (CSR), modifying their BCR specificities and differentiating into either memory B cells expressing surface IgG, IgA, or IgE, or long-lived plasma cells secreting class-switched immunoglobulins (39). While plasma cells are numerous and short-lived, memory B cells are long-lived and less abundant (12). B-lineage transcription factors repress plasma cell differentiation; thus, they must be silenced to initiate the plasma cell program. BCL6 prevents premature plasma cell differentiation by repressing genes involved in BCR signaling cascades, as well as the master regulators of plasma cell fate, such as BLIMP-1 and IRF4. Furthermore, BCL6-mediated repression of BCL2 sensitizes GC B cells to apoptosis, eliminating low-affinity clones. Consequently, BCL6 is considered the central regulator of the GC reaction (39).

Only at the terminal stages of B cell immune responses—when the B cell silences lineage-specific genes and transitions into an antibody-secreting plasma cell—are EBF1 and Pax5 downregulated (61). BLIMP-1 acts to shut down the B cell and GC programs by repressing Pax5 and BCL6. This repression enables GC B cells to exit the cell cycle and silence genes encoding BCR components and downstream signaling molecules. XBP1 regulates the late stages of plasma cell differentiation by managing the unfolded protein response, which is essential for antibody secretion. Additionally, the development of long-lived plasma cells and efficient GC reactions depend on IL-21 receptor-mediated activation of STAT proteins. BCR or CD40 signaling enhances IL-21R expression, and IL-21 binding promotes CD4-dependent activation, proliferation, and CSR (preferentially to IgG), culminating in the differentiation of B cells into plasma cells (39).

### 3.4 Immunological mechanisms of tolerance and the role of transcriptional factors

The normal immune system displays a considerable degree of autoreactivity; therefore, effective tolerance mechanisms are essential to suppress autoreactive cells and prevent their progression into pathogenic effector cells, which could lead to autoimmune diseases (113). Immune tolerance is defined as the permanent restriction of potentially harmful immune responses to innocuous stimuli (114).

Tolerance checkpoints are classified as either central or peripheral, depending on their anatomical location and the stage of B cell differentiation at which they occur (113). Central tolerance occurs in undifferentiated B cells within the bone marrow when they express surface IgM-class antigen receptors but are not yet fully mature. This mechanism is primarily mediated through clonal deletion, anergy, and receptor editing (115). Clonal deletion involves the apoptotic elimination of cells that bind multivalent membrane-bound antigens or that express non-functional BCRs. Anergy refers to the functional inactivation of B cells that chronically bind soluble self-antigens, typically marked by downregulation of BCR expression (116). Anergic B cells exhibit impaired PI3K signaling and disrupted metabolic reprogramming in response to BCR or TLR4 stimulation (117). Receptor editing is the process by which new rearrangements occur, primarily in the light chain (L chain) loci—and less frequently in the heavy chain (H chain)—resulting in a modified BCR with different antigen specificity (116).

In general, B cell immune tolerance is established through the elimination of autoreactive B cells, receptor editing, or reductions in their survival and function. This regulation decreases the frequency of autoreactive B cells in the overall repertoire, as well as their affinity for self-antigens and their capacity to mediate immune responses. After exiting the bone marrow, immature B cells migrate to secondary lymphoid organs (SLOs), where additional tolerance mechanisms are implemented (114). Despite the efficacy of central tolerance, autoreactive B cells can still be found in transitional and naïve B cell compartments; thus, further regulation occurs through peripheral tolerance mechanisms at later stages of differentiation (113).

The maintenance of immunological tolerance in B cells is orchestrated by a complex network of transcription factors, the dysregulation of which can lead to autoimmune manifestations. For instance, Ikaros (IKZF1) promotes anergy by upregulating genes that inhibit BCR signaling and downregulating the TLR/MyD88–NF-κB pathway, thereby preventing the activation of autoreactive B cells. Deficiency in Ikaros leads to systemic autoimmunity in murine models (118). PU.1 (SPI1), an ETS family transcription factor, also contributes to tolerance by regulating functional pathways in B cells, monocytes, and T cells. Reduced PU.1 expression, which can be induced by miR-155 in inflammatory conditions such as rheumatoid arthritis, is associated with enhanced autoantibody production (119, 120). In contrast, premature expression of Blimp-1 (PRDM1) during early B cell development promotes the differentiation of autoreactive plasmablasts, ultimately triggering autoantibody production and glomerulonephritis in mouse models (121). Together, these findings highlight the pivotal roles of Ikaros as a tolerogenic suppressor, PU.1 as a regulator of B cell identity and function, and Blimp-1 as a potentially pathogenic factor when deregulated—all components of a delicate transcriptional balance critical to the prevention of autoimmunity.

A subset of B cells characterized by CD11c expression, T-bet transcription factor expression, and lack of CD21 and CD23 expression has been implicated in the pathogenesis of systemic lupus erythematosus (SLE). Known as DN2 cells in humans, this subset is induced by a combination of disease-elevated stimuli, such as TLR7 ligands, interferon-gamma (IFN-γ), and interleukin-21 (IL-21) (122). Once fully differentiated, DN2 cells exhibit heightened sensitivity to TLR7 signaling and are predisposed to develop into plasma cells that secrete autoantibodies, including anti-dsDNA antibodies commonly associated with SLE (123). Upon encountering antigens, autoreactive follicular (FO) B cells upregulate T-bet, differentiate into age-associated B cells (ABCs), and eventually become plasma cells—major contributors to IgG autoantibody production. While T-bet is not strictly required for aberrant plasma cell differentiation or autoantibody production, its expression marks B cells that have undergone pathogenic differentiation, making it a valuable marker of autoreactive B cells rather than a prerequisite for their emergence (124).

Beyond the core transcription factors directly involved in anergy and immune suppression, several additional regulators—including ZEB2, ETS1, LEF1, EBF1, and E2A—also influence the immunological landscape by modulating the functional repertoire, activation potential, and autoreactive tendencies of B cells. For example, ZEB2 has been identified as a key driver of age-associated B cells (ABCs), a proinflammatory subpopulation that expands in autoimmune conditions and aging, contributing to the breakdown of peripheral tolerance (125). ETS1, whose expression is decreased in autoimmune diseases such as lupus, acts as a negative regulator of autoreactive plasma cell differentiation. Its low expression, along with that of LEF1, in the salivary glands of patients with Sjögren’s syndrome suggests a permissive microenvironment for persistent lymphocyte activation (126, 127). EBF1 regulates the expression of costimulatory genes such as SLAMF1, whose overexpression may enhance pathological interactions with T cells (128). Although E2A is primarily studied in T lymphocytes, it remains essential for maintaining B cell identity and may indirectly influence humoral tolerance. While these transcription factors do not directly mediate classical tolerance mechanisms like clonal deletion or anergy, their roles in shaping B cell phenotype and function create immunological conditions that may favor or impair self-tolerance.

## 4 Conclusions

B cell development is a dynamic and tightly regulated process involving intricate networks of transcription factors, microenvironmental cues, and immune tolerance mechanisms. Each stage of differentiation—from hematopoietic stem cell to plasma or memory B cell—relies on the coordinated expression of key genes such as *PU.1, Ikaros, Pax-5, BLIMP-1*, and *BCL6*, along with the precise integration of antigenic and costimulatory signals. Disruptions in this regulation, whether through genetic mutations, epigenetic alterations, or chronic inflammatory states, can lead to autoimmune diseases, immunodeficiencies, or B cell neoplasms.

A deeper understanding of the transcriptional and immunological mechanisms that govern B cell ontogeny and function provide new opportunities for the development of targeted therapies. Identifying critical elements involved in tolerance breakdown or resistance to immunotherapeutic interventions will support the design of more precise and personalized treatment strategies for autoimmune and lymphoproliferative disorders. Moreover, the integration of emerging technologies such as single-cell transcriptomics and gene editing offers promising tools to further elucidate key events in B cell biology with unprecedented resolution.
